# Neural underpinning of Japanese particle processing in non-native speakers

**DOI:** 10.1038/s41598-022-23382-8

**Published:** 2022-11-05

**Authors:** Chise Kasai, Motofumi Sumiya, Takahiko Koike, Takaaki Yoshimoto, Hideki Maki, Norihiro Sadato

**Affiliations:** 1 Faculty of Regional Studies, Gifu University, Yanagido, 501-1193 Japan; 2 Research Center for Child Mental Development, Hamamatsu University School of Medicine, Hamamatsu, 431-3192 Japan; 3 Division of Cerebral Integration, National Institute for Physiological Sciences, Okazaki, 444-8585 Japan; 4 Department of Physiological Sciences, SOKENDAI (The Graduate University for Advanced Studies), Hayama, 240-0193 Japan; 5 Research Organization of Science and Technology, Ritsumeikan University, Kusatsu, 525-8577 Japan

**Keywords:** Psychology, Human behaviour

## Abstract

Grammar acquisition by non-native learners (L2) is typically less successful and may produce fundamentally different grammatical systems than that by native speakers (L1). The neural representation of grammatical processing between L1 and L2 speakers remains controversial. We hypothesized that working memory is the primary source of L1/L2 differences, by considering working memory within the predictive coding account, which models grammatical processes as higher-level neuronal representations of cortical hierarchies, generating predictions (forward model) of lower-level representations. A functional MRI study was conducted with L1 Japanese speakers and highly proficient Japanese learners requiring oral production of grammatically correct Japanese particles. We assumed selecting proper particles requires forward model-dependent processes of working memory as their functions are highly context-dependent. As a control, participants read out a visually designated mora indicated by underlining. Particle selection by L1/L2 groups commonly activated the bilateral inferior frontal gyrus/insula, pre-supplementary motor area, left caudate, middle temporal gyrus, and right cerebellum, which constituted the core linguistic production system. In contrast, the left inferior frontal sulcus, known as the neural substrate of verbal working memory, showed more prominent activation in L2 than in L1. Thus, the working memory process causes L1/L2 differences even in highly proficient L2 learners.

## Introduction

Although a growing body of research has been investigating the similarities and differences in linguistic processing between native (L1) and non-native (L2) speakers, the issue still remains controversial. Some studies have shown that the acquisition of grammar by late learners is typically less successful and produces less uniform, and perhaps even fundamentally different grammatical systems than with L1 acquisition^1,2^. These studies argue that the rule systems developed by late L2 learners do not necessarily conform to the principles that constrain native grammar learners^2–5^. Conversely, evidence from several experimental studies indicates that late L2 learners can achieve native-like processing^6–9^.

Two principal hypotheses have been expounded regarding L2 grammatical processing of sentence comprehension. Clahsen and Felser’s^4,5^ influential shallow structure hypothesis postulates that L2 learners adopt “shallow” parsing with reduced sensitivity to grammatical information; thus, a different parsing process occurs from L1. The second hypothesis assumes that L1/L2 parsing processing is similar and explains the differences therein, in terms of inefficient lexical access routines or an increased burden on capacity-limited cognitive resources such as working memory in L2^10–12^. By specifying working memory function as memory retrieval, Cunnings^13^ argued that “a primary source of L1/L2 differences (in the grammatical process) lies in the ability to retrieve information-constructed processing from memory”.

Working memory involves holding information in the mind and mentally working with it^14^. "Working memory is critical for making sense of anything that unfolds over time, for that always requires holding in mind what happened earlier and relating that to what comes later. Thus, it is necessary to make sense of written or spoken language, whether it is a sentence, a paragraph, or longer"^14^. Working memory is a core executive function and is defined as "a collection of top-down control processes used when going on automatic or relying on instinct or intuition would be ill-advised, insufficient, or impossible"^14^. Other core executive functions are inhibition and cognitive flexibility^14^.

One approach to studying the issue of L1/L2 differences is by using a functional neuroimaging technique, such as functional MRI (fMRI) or PET. Previous studies have indicated that L2 syntax requires a high processing load and is represented in language-related areas^8,15^. For L1, the neural substrates of grammatical processing comprise the frontal and posterior superior temporal regions and their connections via dorsal fiber pathways^16–23^. However, recent neuroimaging and lesion studies suggest that grammar is inseparable from other aspects of language comprehension (lexico-semantic processing)^24,25^. Thus, the study of L2 grammatical processing of sentence comprehension requires a procedure that is able to investigate and manipulate the grammatical component within sentence comprehension.

In this study, we investigated the mechanism of L2 grammar processing using Japanese particles. Japanese is a head-final language with a subject-object-verb (SOV) word order, in which, particles are crucial in providing a thematic role to nouns within the sentence (e.g., agent, patient, location, goal)^26,27^. A particle is a suffix represented by a Japanese hiragana character and is normally added to the end of a noun. As the head (verb) is not stated until the end of the sentence, Japanese sentences are incrementally processed before the head is inputted^28,29^. As the information contained in a particle affects the prediction or anticipation of subsequent elements^30,31^, selecting the correct particle is essential for creating a comprehensible and grammatically acceptable sentence in Japanese. To articulate such a sentence, speakers must select the appropriate particle. However, the treatment of particles is difficult. Unlike English, in which the word order determines the grammatical role of the noun in a sentence, in Japanese, the grammatical function is signaled through postpositional particles^32^. For example, by adding the nominative case particle *ga,* the noun becomes a subject, and the accusative particle *o* indicates that a noun is an object in a sentence. Although Japanese is an SOV language, the order is free because the word order is not essential for assigning grammatical roles. Thus, a speaker needs to assign a correct particle to assign a thematic role to the nouns. What makes particle selection especially difficult for L2 learners is that the same particle is used for different purposes. For example, *wa* has two functions: to mark a theme or to mark a contrasting element in a sentence^33^. Furthermore, in a colloquial form, particles are often omitted^34^. The lower the level of formality, the more acceptable the particle omission is^35^, and particles can be dropped when adjacent to a verb^36^. Although numerous studies have investigated particle omission, not much is known about which particle is omitted the most. Such context-dependent operations make L2 learning more difficult. Inaccurate use of particles is seen among both Japanese children^37^ and proficient learners with years of language-learning experience^38,39^. Thus, correct particles usage is a known bottleneck for non-native learners, and are simultaneously well-suited to experimentally control the grammatical structures; further, minimal effort is required to replace or omit them since they are represented with one mora.

The neural substrates of particle processing were first examined by Inui et al.^40^ in a discrimination task in which they showed particles and non-particles without any other sentence information to native Japanese participants (e.g., *X ga* (particle) or *X nu* (non-particle)) and asked to judge whether they were particles. They compared the results with those from a phonological discrimination task to conclude that the left inferior frontal gyrus (IFG) was responsible for particle discrimination in Japanese. While the involvement of the left IFG in particle processing has also been reported by other researchers^41,42^, they did not test the processing of the retrieval and selection of an appropriate particle, which is relevant for communication. Furthermore, the neural underpinnings of particle processing by non-native Japanese learners have never been reported.

**Figure 1:**
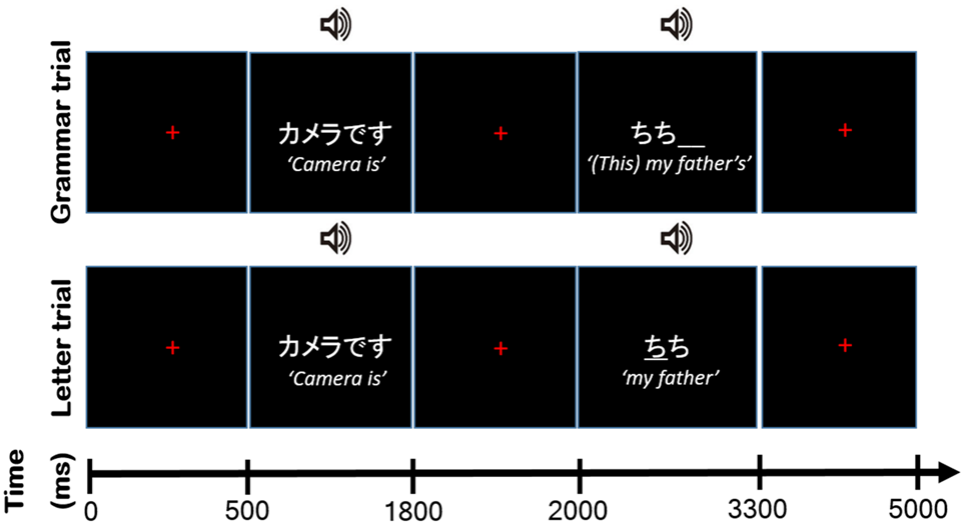
Sequence of events in the task. Each trial comprised two phases: preparation and production. In the preparation phase, the participant observed and listened to the predicate portion (*Camera is*). In the production phase, the subject *(This) my father* was presented. Two conditions were prepared in this phase: when an empty line was shown, the participant was required to utter a particle (Grammar condition). In this particular case, *no* (the possessive case) was the correct answer. When a Japanese letter was presented with an empty line, the participant was required to read out the letter (Letter condition). Activities during the task were modelled with boxcar functions for each phase, except for the rest condition. The English phrases in the figure is for explanatory purposes, and were not presented during the experiment.

The current study attempted to fill the gaps in previous studies and aimed to test two contradicting hypotheses on L2 grammatical processing. We recruited highly proficient L2 Japanese learners of various nationalities residing in Japan, whose ability to produce particles was tested; native Japanese speakers comprised the L1 control group. Highly proficient L2 participants were chosen as a proof that Japanese particles are bottle necks for learning the Japanese language. All participants were assigned a sentence completion task in which they were instructed to select an appropriate particle (Fig. 1) (Grammar condition) orally and to simply read out an underlined Japanese phonetic letter (Letter condition). Unlike the previous studies, we aimed to examine the process of the actual language production.

We hypothesized that working memory is a primary source of L1/L2 differences in the grammatical process^13^. Specifically, we operationalized working memory and executive function within the predictive coding account, which is the most popular explanation for neuronal message passing^43–46^. In this account, neuronal representations in higher levels of cortical hierarchies generate predictions (forward model) of representations in lower levels^44,45,47^. The comparison of top-down predictions with representations at the lower level forms a prediction error that is passed back up the hierarchy to update higher representations. This recursive exchange of signals suppresses the prediction error at each level and provides a hierarchical explanation for sensory inputs that enter at the lowest (sensory) level. The neuronal activity of the entire hierarchy encodes beliefs (or probability distributions) over states in the world that cause sensations (e.g., my visual sensations are (likely) caused by a face)^48^. Thus, *perceptual inference* is accomplished by minimizing prediction errors by changing the top-down prediction. *Active inference*, in contrast, minimizes prediction errors by changing sensory inputs through action, such as utterance^49^. According to a theoretical analysis of the relationship between working memory and active inference, working memory is considered a process of evidence accumulation to inform action choices through active inferences^50^**.** In the current study, we asked participants to select the most suitable Japanese particle to fulfill a blank in a stimulus sentence, which is a working memory process.

Participants were given the following phrases, serially (Fig. 1),1

They had to re-order the phrases mentally,2 and utter the most appropriate particle in the context (2), that makes grammatical sense.3

The comparison of top-down predictions (forward model) with representations at the lower level (Sentence (3)) forms a prediction error. The exploration of particles for active inferences continues until the minimum prediction error is reached. Thus, according to predictive coding theory, working memory includes the active inference process that minimizes prediction error. This formalization first enables the plausible interpretation of the behavioral differences between L1 and L2: a longer RT represents more iterative processing to minimize the prediction error. Second, it provides the explicit hypothesis regarding the neural representation of L1/L2 difference: our working memory hypothesis predicts the activation difference of L1/L2 in the verbal working memory region. Third, this formalization shows how immersion works for learning L2: optimization of the forward model can be accomplished by sharing the forward model through mutual interaction with active inference^48^. Specifically, working memory in the present task consists of.

- Mentally holding the serially presented phrases, (1)
- Re-ordering them, (2)
- Retrieving the particle according to a prior probability distribution, (3)
- Comparison of (3) with a forward model to generate the prediction error.

The whole process from (a) through (d) is regarded as working memory, leading to the decision to move on to the second round by inhibiting the preceding results to explore other candidate's particles. Thus, the active inference is one of the possible mechanisms of working memory, that is, minimizing the prediction error, leading to the decision process that depends on other executive functions such as inhibition and cognitive flexibility.

Within this predictive coding schema, the forward model of L2 is assumed to be less optimized than that of L1; we hypothesized that L2 requires a longer reaction time (RT), and more workload on the working memory is reflected by the more prominent activation of the verbal working memory region in the left inferior frontal sulcus. This hypothesis derives from the fact that the lexical processes of L1 are almost automatic whereas those of L2 require effort, implying the recruitment of the executive function. This difference corresponds to the prediction error in the predictive coding schema: the smaller the error, the smaller the number of iterations of active inference to minimize it. Within the context given by the two phrases, the optimal forward model of L1 weighted a few candidates of the particle from a variety of contextual options. In contrast, a less optimal forward model of L2 cannot limit the candidates’ number of particles, resulting in longer reaction times. If the shallow structure hypothesis (postulating L2 learners adopt “shallow” parsing with reduced sensitivity to grammatical information—a different parsing process from L1) is correct, activation patterns in the particle-related area and the left IFG^40^ will differ, and the working memory-related areas will show similar activation patterns.

In the present study, we conducted an fMRI experiment involving 23 healthy non-native learners of Japanese and 25 healthy native speakers among Japanese adult volunteers. We employed a task in which each participant was required to read out a particle by filling in a blank or reading a letter on the screen within the MRI scanner. To investigate our hypotheses, we compared the RTs and error rates used to produce particles or letters between native and non-native participants, and the associated neural processing by focusing on similarities and differences.

## Results

### Behavioral results

**Figure 2:**
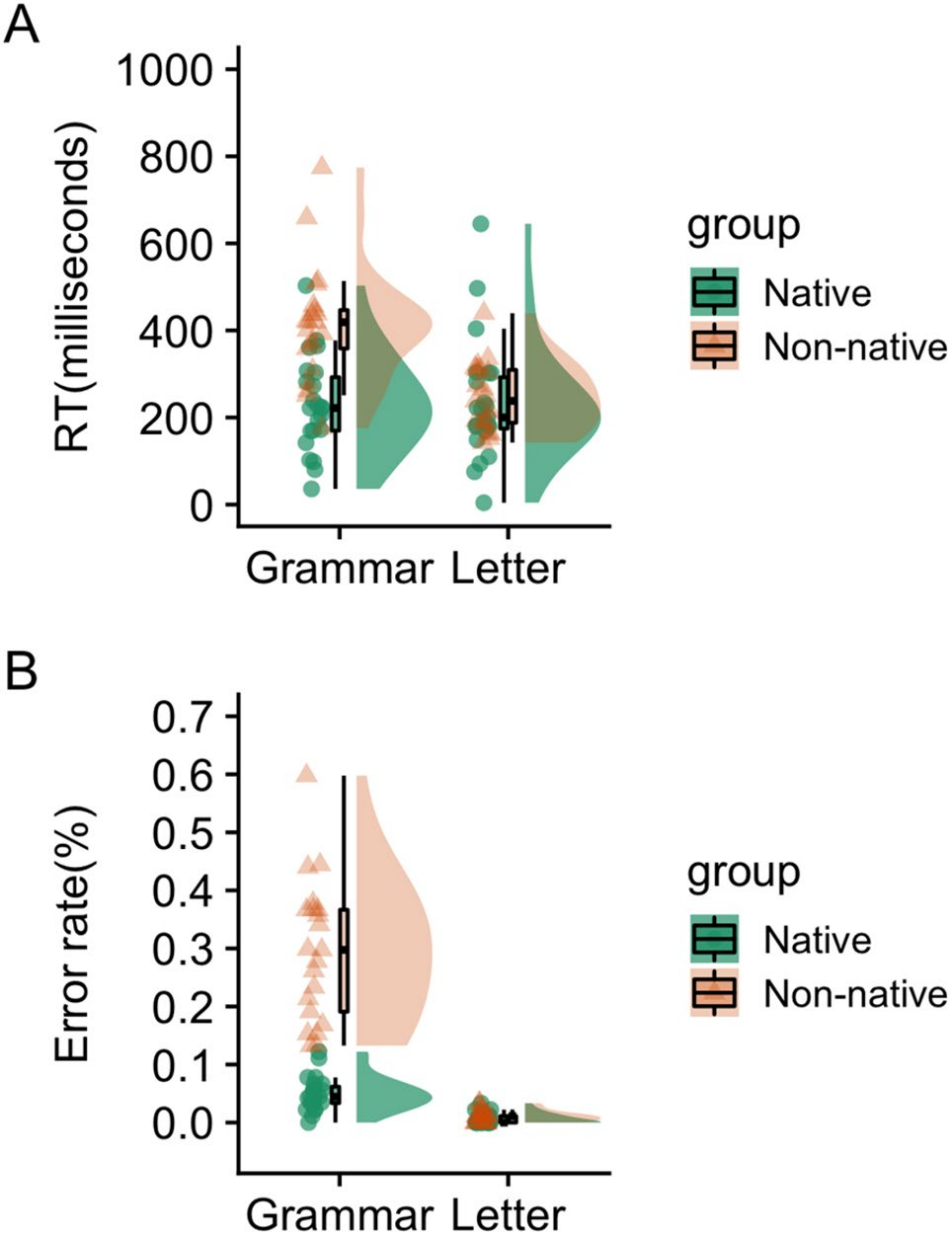
Behavioral results. Non-native learners demonstrated significantly (**A**) longer reaction times and (**B**) higher error rates than native speakers. Data are presented as box plots and violin plot datasets for each group.

We measured the RT of the produced particles and letters, and eliminated RTs from further analysis of two native participants whose raw RTs in both conditions were longer than 2 standard deviations (SDs) from the native group average. This was done as longer RTs indicated misunderstanding of the task (N = 23; total in the non-native learners’ group). We also eliminated from further analysis, the data of one non-native participant whose utterance could not be recorded due to a recording error (N = 21; total in the native learners’ group). The results of the linear mixed-effects modelling for RT indicated that the interaction between conditions and groups was significantly associated with performance (estimate = 0.12, SE = 0.017, t = 6.874). There was also a significant main effect of conditions (estimate = 0.056, SE = 0.011, t = 4.938) and groups (estimate = 0.071, SE = 0.025, t = 2.903) (Supplementary Table S5 and Fig. 2A). In addition to Fig. 2A, which shows raw values of RT, the figure showing the log-transformed RT is shown in Supplementary Figure S1.

### Error rate

We checked the error rate in the produced particles and letters. Results of linear mixed-effects modelling for accuracy indicated that the interaction of conditions and groups was significantly related to performance (estimate = −2.406, SE = 0.607, t = −3.963). There was also a significant main effect of conditions (estimate = −3.151, SE = 0.34, t = −7.905) and groups (estimate = −2.305, SE = 0.392, t = −5.886) (Supplementary Table S5 and Fig. 2B).

All graphs were prepared using the RainCloudPlots R-script^51^, which provides a combination of box, violin, and dataset plots. In the dataset plot, each dot represents a data point. In the boxplot, the line dividing the box represents the median of the data, while the ends of the box represent the upper and lower quartiles. The extreme lines show the highest and lowest values excluding outliers.

### Correlation between immersion length and behavioral performance

To investigate the relationship between the performance and the contribution of experience of staying or living in Japan, we conducted the correlation analysis between each participant’s full-immersion duration and performance in the non-native group. We found that, as the length of stay in Japan increased, a participant’s error rate significantly decreased (r = −0.52, *p* = 0.015), although there was no such significant relationship in the RT (r = 0.22, *p* = 0.328).

### Functional MRI results

**Figure 3:**
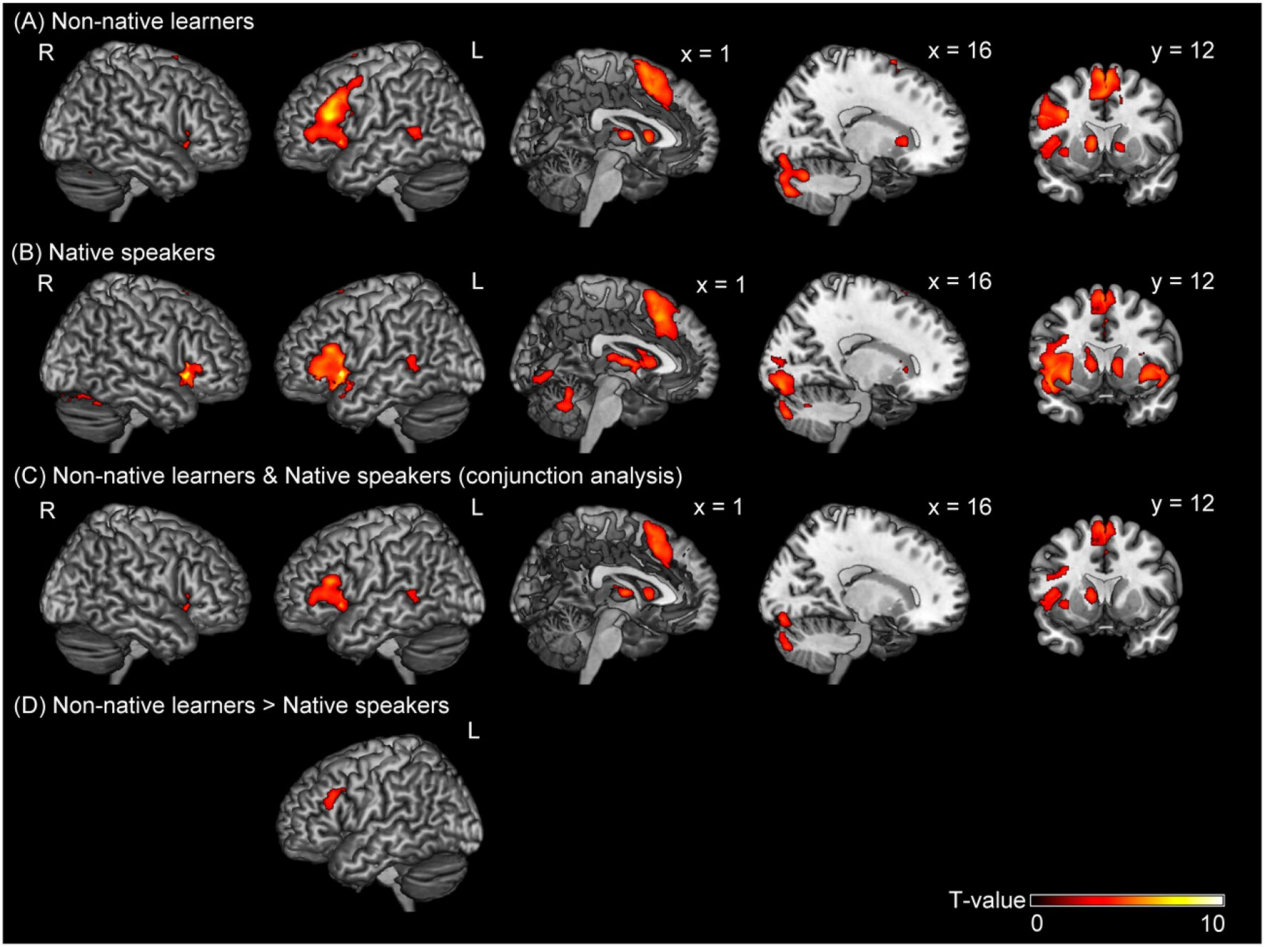
Regions associated with correct grammar processing. Brain activation was associated with grammar processing (grammar > letter) in (**A**) non-native learners, (**B**) native speakers, (**C**) the conjunction of non-native learners and native speakers, (**D**) non-native learners greater than native speakers, and (**E**) native speakers greater than non-native learners. The level of activation was set at a threshold *p*-value of < 0.05 and the FWE was corrected for multiple comparisons over the whole brain, with the height threshold set at a *p*-value of < 0.001 (uncorrected).

The whole brain analysis with contrasts of (grammar > letter) revealed significant activation in the bilateral IFG/insula and superior frontal gyrus (SFG) corresponding to the supplemental motor area/middle cingulate cortex, caudate, left middle frontal gyrus (MFG)/precentral gyrus, left middle temporal gyrus, and bilateral cerebellum in non-native learners (Fig. 3A and Table 1) and in the bilateral IFG/insula, SFG (supplemental motor area)/middle cingulate cortex, caudate, left middle temporal gyrus, and bilateral cerebellum in native speakers (Fig. 3B and Table 1).

**Table 1:** Regions associated with processing in non-native learners and native speakers.

Spatial extent test		MNI coordinates (mm)			t value	Location	
Cluster size	PFWE-corr	x	y	z		Side	Area
**In non-native learners**							
13,387	< 0.001	−3	8	64	5.95	L	SFG
		1	9	63	6.18	R	SFG
		− 5	18	44	5.56	L	mPFC
		14	25	33	5.72	R	MCC
23,272	< 0.001	− 34	−1	45	4.83	L	Precentral gyrus
		− 54	28	19	8.27	L	IFG, triangular
		− 46	15	6	5.92	L	IFG, opercular
		− 35	19	− 1	6.75	L	Insula
		− 33	35	− 2	5.12	L	IFG, orbital
829	0.022	− 3	− 15	13	3.70	L	Thalamus
5112	< 0.001	40	26	7	4.97	R	IFG, triangular
		13	17	3	5.17	R	Caudate
		35	24	− 1	5.96	R	Insula
		35	32	− 5	4.07	R	IFG, orbital
		26	19	6	4.32	R	Putamen
3748	< 0.001	− 50	− 41	7	4.99	L	MTG
1013	0.008	− 11	12	7	5.54	L	Caudate
		− 11	6	0	4.77	L	Pallidam
704	0.045	51	− 31	3	4.75	R	STG
10,800	< 0.001	18	− 85	− 14	4.77	R	Lingual gyrus
		20	− 67	− 27	5.44	R	Cerebellum VI
		14	− 65	− 29	5.29	R	Cerebellum Dentate
		41	− 61	− 30	4.76	R	Cerebellum Crus I
		11	− 83	− 30	6.19	R	Cerebellum Crus II
		27	− 65	− 46	6.74	R	Cerebellum VIIb
**In native speakers**							
15,126	< 0.001	− 9	21	46	6.50	L	SFG
		1	26	39	5.06	R	mPFC
		− 3	26	38	5.29	L	MCC
		14	20	33	6.30	R	MCC
		− 12	28	29	4.97	L	ACC
21,757	< 0.001	− 55	27	18	6.31	L	IFG, triangular
		− 47	14	6	6.34	L	IFG, opercular
		− 33	19	− 1	9.63	L	Insula
		− 46	17	−8	5.88	L	IFG, orbital
		− 46	20	− 15	5.83	L	Temporal pole
12,286	< 0.001	38	19	13	4.55	R	IFG, opercular
		36	32	4	5.29	R	IFG, triangular
		37	22	− 3	8.02	R	Insula
		48	24	− 10	4.79	R	IFG, orbital
5067	< 0.001	3	− 8	8	5.70	R	Thalamus
		− 11	9	5	4.82	L	Caudate
		11	13	4	4.93	R	Caudate
7592	< 0.001	12	− 89	7	4.99	R	Calcarine gyrus
		− 1	− 79	− 7	4.29	L	Lingual gyrus
		21	− 87	− 13	5.92	R	Lingual gyrus
		13	− 82	− 34	5.95	R	Cerebellum Crus II
1714	< 0.001	− 57	− 37	5	4.88	L	MTG
1448	< 0.001	0	− 56	− 21	5.03		Cerebellum I−IV
		5	− 55	− 26	4.52	R	Cerebellum Fastigial
		21	− 68	− 26	3.43	R	Cerebellum VI
		1	− 61	− 29	4.54	R	Cerebellum Vermis VI
		9	− 64	− 30	4.32	R	Cerebellum Dentate
		− 1	− 59	− 32	4.38	L	Cerebellum Vermis VIIIa
744	0.036	34	− 67	− 23	3.95	R	Cerebellum VI
		45	− 57	− 29	4.04	R	Cerebellum Crus I

Activation was set at a threshold of p < 0.05 and FWE was corrected for multiple comparisons over the whole brain, with the height threshold set at p < 0.001, uncorrected. The terms x, y, and z represent the stereotaxic coordinates (mm). R, right hemisphere; L, left hemisphere; IFG, inferior frontal gyrus; SFG, superior frontal gyrus; ACC, anterior cingulate cortex; MCC, middle cingulate cortex; mPFC, medial prefrontal cortex; MTG, middle temporal gyrus; FWE, family-wise error; MNI, Montreal Neurological Institute.

The conjunction analysis with the contrasts of (grammar > letter) in native speakers and non-native learners revealed significant activation in the bilateral IFG/insula, left SFG (supplemental motor area)/middle cingulate cortex, left caudate, left middle temporal gyrus, and right cerebellum (Fig. 3C and Table 2).

**Table 2:** Regions associated with grammar processing for non-native learners and native speakers.

Spatial extent test		MNI coordinates (mm)			t value	Location	
Cluster size	*P*_FWE-corr_	x	y	z		Side	Area
**Non-native learners and Native speakers (conjunction analysis)**							
9040	< 0.001	2	14	60	5.26	R	SFG
		−4	19	45	5.51	L	SFG
		14	25	33	5.72	R	MCC
13,334	< 0.001	−55	27	18	6.31	L	IFG, triangular
		−46	15	6	5.92	L	IFG, opercular
		−35	19	−1	6.75	L	Insula
		−33	35	−2	5.12	L	IFG, orbital
711	0.043	−3	−15	13	3.70	L	Thalamus
4416	< 0.001	40	26	7	4.97	R	IFG, triangular
		35	24	−1	5.96	R	Insula
		35	32	−5	4.07	R	IFG, orbital
		26	19	−6	4.32	R	Putamen
879	0.016	−11	9	5	4.82	L	Caudate
		−11	6	1	4.60	L	Pallidum
1308	0.002	−54	−36	5	4.56	L	MTG
1764	< 0.001	18	−85	−14	4.77	R	Lingual gyrus
		20	−79	−20	3.46	R	Cerebellum VI
		17	−86	−28	3.84	R	Cerebellum Crus I
		13	−83	−32	5.32	R	Cerebellum Crus II

Activation was set at a threshold value of *p* < 0.05 and FWE was corrected for multiple comparisons over the whole brain, with the height threshold set at *p* < 0.001, uncorrected. The terms x, y, and z represent the stereotaxic coordinates (mm). R, right hemisphere; L, left hemisphere; IFG, inferior frontal gyrus; SFG, superior frontal gyrus; MCC, middle cingulate cortex; MTG, middle temporal gyrus; MNI, Montreal Neurological Institute.

In the comparison of (grammar > letter), non-native learners showed higher activation in the cluster including the left IFG and MFG on the left inferior frontal sulcus (LIFS) (Fig. 3D and Table 3) than native speakers.

**Table 3:** Regions associated with more grammar processing in non-native learners or native speakers.

Spatial extent test		MNI coordinates (mm)			t value	Location	
Cluster size	PFWE-corr	x	y	z		Side	Area
**Non-native learners > Native speakers**							
752	0.034	−52	15	37	3.40	L	MFG
		−52	25	31	4.62	L	IFG, opercular

Activation was set at a threshold value of *p* < 0.05 and FWE was corrected for multiple comparisons over the whole brain, with the height threshold set at *p* < 0.001, uncorrected. The terms x, y, and z represent the stereotaxic coordinates (mm). R, right hemisphere; L, left hemisphere; IFG, inferior frontal gyrus; MFG, middle frontal gyrus; MNI, Montreal Neurological Institute.

## Discussion

In this study, we compared the role of working memory processes among native and highly proficient adult learners of Japanese. In terms of behavior, as expected, we found a longer RT and a higher error rate in non-native learners than in native speakers when producing particles. According to predictive coding theory, a shorter RT in native speakers (Fig. 2B) reflects faster processing of minimizing the prediction error. This may be related to the forward model formation optimized to the context generated by the serially presented phrases. This process may correspond to the context-dependent anticipation of correct particle candidates. Cunnings^13^ argued that the primary difference in sentence processing between L1 and L2 was in the ability to retrieve lexical information, known as lexical semantics, from memory. Thus, the differences may suggest that non-native learners acquired sufficient lexical semantics to select particles correctly. Native speakers use a combination of the lexical semantics of a noun phrase and its particles to anticipate upcoming arguments without awaiting the arrival of the verb^52^. As a forward model formation corresponds to the Bayesian inference with the prior probability^53^, native speakers may predict the correct particles' probability using a combination of the retrieved lexical semantics. Several studies have suggested that L2 learners do not anticipate the upcoming arguments to the same extent as native speakers do^54–56^. This does not necessarily mean they exhibit a lack of lexical semantics because the present study participants were highly proficient. Instead, the context-dependent forward model formation with lexical semantics of L2 may not be as efficiently conducted as that of L1s. This notion is supported by the findings that the staying experience of L2 in Japan significantly decreased the error rate. This finding is consistent with the active inference account of communication. Friston and Frith^57^ argued that communication facilitates long-term changes in the interacting individuals' forward models by predicting and minimizing their mutual prediction errors if these agents adopt the same forward model. Thus, the longer the stays within the areas where the L2 is publicly used, the better adjusted the forward model is, resulting in lower error rates.

As for different brain regions for particle processing, when comparing non-native learners to native speakers, the LIFS was more prominently activated. Note that the task difficulty of each test item within the group was modelled (Fig. 3D). Thus, the differences in neural activation reflected differences in the processing of sentence-building by selecting an appropriate particle between L1 and L2 participants, rather than its difficulty. Furthermore, we focused on correct trials, excluding the incorrect response trials from the analysis. Thus, it is unlikely that the additional brain regions recruited in the L2 group were related to error processing.

Several neuroimaging studies have reported separate functions within the inferior frontal cortex^58,59^. As a core region for grammar, the left IFG, and for non-grammar working memory, the LIFS, are dissociated from each other within the inferior frontal cortex. For example, in the study by Makuuchi et al*.*^59^, an embedded sentence structure was utilized to examine how multiple subject-noun phrases were stored before being processed. The results provide direct evidence of dissociation, with the left pars opecularis (LPO) associated with core grammatical computation and the LIFS associated with non-grammatical working memory. This finding was consistent with a meta-analysis that determined that the mid-lateral prefrontal cortex clustering in and around the IFS is a core region of the executive component of working memory^60^. Considering the slower response rate and lower accuracy for grammatical processes among non-native learners in the present study, this group may have to select the correct particle by going through multiple choices among all available particles, whereas native speakers have fewer choices in the given context due to the accumulated prior experience and thus the choices are relatively less complex. Although we did not directly show that L2 speakers went through multiple choices, through the predictive coding schema, the elongated RT can be explained by the increased iteration of the retrieval. Thus, the enhanced neural activity of the LIFS can be interpreted as the reflection of a larger prediction error and more iterations of the retrieval. This is consistent with experience-based theories of the complexity difference of grammatical processes^61–65^, and our predictive coding account. Therefore, for non-native learners, the activation of the LIFS may be associated with a greater workload on the working memory recruited for minimizing prediction errors between the forward model and the retrieved particles. The LIFS is related to the post-retrieval selection in resolving competition between simultaneously active representations^60,66^.

In terms of common brain regions for particle processing, as compared to letter processing, the bilateral IFG/insula, SFG/middle cingulate cortex, left caudate, left middle temporal gyrus, and right cerebellum were activated in both native speakers and non-native learners, while selecting and uttering particles (Fig. 3C) that required syntax computation and speech production. Our data confirmed that similar brain areas are recruited for particle production as for grammatical processing^67^. The activation pattern is largely consistent with previous literature indicating that these brain areas are fundamental to the processing of syntax production^68,69^.

Regarding grammatical processing, the previous studies are in agreement that the left IFG is particularly the core region^69–71^ for hierarchical grammatical complexity^72^. Therefore, the activation of the left IFG for particle processing could be interpreted by assuming a cognitive process that is routinely engaged when attempting to comprehend linguistic input that is relatively challenging to understand due to its grammatical properties^69^.

Furthermore, although little activation of the right IFG has been reported for grammatical processing^69^, its co-activation with the left IFG is observed in tasks that reflect linguistic expectations, that is, the prediction of structural features of the expected linguistic input^73–75^. Therefore, in our task, which provides linguistic expectation and allows for retrieval of the particle as soon as the noun is presented, bilateral activation of the IFG may be associated with grammatical surprise, reflecting the expectedness of the particle given its preceding context, which allows the noun to be connected to the left context^74^.

The activation of bilateral IFG included the activation of the bilateral insula within these clusters. A previous activation likelihood estimation (ALE) meta-analysis reported the involvement of the bilateral insula for speech and language tasks^76^. For speech production, it has been suggested that the insula cortex functions as a relay between the cognitive aspect of language and preparation for vocalization^77^, particularly during difficult speech-language processing^78^. Therefore, for both native speakers and non-native learners, the bilateral insula’s involvement in grammatical processing is attributed to an integrator of grammatical computation and speech production through cooperation with other regions.

Another common region for native speakers and non-native learners was the pre-SMA, which is known to be involved in word selection, encoding of word form, and control of syllable sequencing^79,80^. Furthermore, the linguistic system relies on a bilateral dual structure^23,81^, while the pre-SMA is involved in the interaction of right- and left-hemispheric function between prosodic representations of speech melody and rhythm in the right hemisphere and the parsing of abstract grammatical constituents in the left hemisphere^82^. Therefore, the pre-SMA may coordinate the activity of the bilateral IFG and insula to process an appropriate particle that needs to be selected quickly. We also found activation in the left caudate and right cerebellum regions. In the planning of speech production, the pre-SMA and basal ganglia such as the caudate is in concert with the cerebellum and serve as a pacemaker to provide a basic temporal structure^80^.

As for the practical implications of the present study, our hypothesis was that proficiency in bilingualism depends on the optimization of the forward model that can be accomplished by sharing it across individuals through mutual communication. Thus, we found that the staying experience within Japan significantly decreased the error rate. This finding is consistent with the active inference account of the communication. Friston and Frith^57^ argued that communication facilitates long-term changes in the interaction among an individual's forward models by predicting that each could minimize their mutual prediction errors if these agents adopted the same forward model. Thus, the longer the stay or experience in the areas where L2 publicly speaks the language, the better adjusted is the forward model and the lower is the error rate. Thus, proficiency in bilingualism may be understood as the optimized forward model^57^ that can be improved by immersion in the L2-spoken region.

This study has some limitations. First, our task was not designed to test the predictive coding hypothesis per se, and thus this study does not depict the complete structure of predictive codings, such as the representation of the forward model which sends the top-down signals to the left IFS nor the lower representation of bottom-up signals. Future study is warranted for the complete depiction of the hierarchical structure of the linguistic process.

In this task, we used seven types of particles containing one mora (one hiragana character), which were selected from case particles, connecting particles, postpositional particles, conjunctive particles, as well as the suffixes of adjectives and adverbs and parts of adverbs. The selection of the particles was based on the Mikiko Iwasaki Systematic Japanese^83^, which focuses on the most effective method of teaching Japanese. Thus, the more frequently the particle is used in our daily life, the more frequently the particle was tested in our experiment, and for this reason, the number could not be controlled. Therefore, presentation of each type of stimuli was not necessarily balanced. Thus, we could not examine differences in RT/error rates across different kind of particles, which may reflect the difficulty associated with each type of particle for non-native participants.

We adopted an explicit particle comprehension task; thus, we could not infer any implicit processes. Within the predictive coding schema, the implicit production of a particle implies applying a forward model without active inference. Thus, we expect that the RT may be similar between L1 and L2. Conversely, the error rate depended on the optimization of the forward model; thus, it was better in L1 than L2 participants. These expectations can be tested in future studies.

In this study, because we did not intend to test any specific hypothesis regarding the L1 effects, the first language of the L2 participants was not controlled and was varied (Table S1). The neural underpinning of L1 effects on L2 performance warrants further study.

In conclusion, the Japanese particle selection task activated the LIFS, known as the neural substrate of verbal working memory, and showed more prominent activation in L2 than in L1 subjects. In contrast, the core linguistic production system of both L1 and L2 subjects was similarly activated. We conclude that the active inference mediated by working memory causes differences in L1/L2 even among highly proficient L2 learners, supporting the working memory hypothesis.

## Methods

### Participants

Twenty-three healthy non-native learners of Japanese aged between 19 and 44 years (9 men and 14 women; mean age = 27.5 years; SD = 5.9 years) and 25 healthy native speakers of Japanese aged between 18 and 39 years (13 men and 12 women; mean age = 23.7 years; SD = 5.4 years) participated in the study (for details, see Supplementary Tables S1and S2).

All subjects gave informed written consent to participate. The present study was approved by the ethics committee of Gifu University and the National Institute for Physiological Sciences and was in accordance with the Declaration of Helsinki. All non-native learners who participated in the experiment had different demographic background on self-reporting such as first language or length of stay in Japan (Supplementary Table S1). All non-native learners had high Japanese proficiency. The majority had passed N1 level of the Japanese Proficiency Test (JLPT), which is equivalent to the Common European Framework of Reference for Language (CEFR) C1 level. One participant had achieved the N2 level (CEFR B2), and another had achieved the N3 level (CEFR B1). One participant undertook the Examination for Japanese University Admission (EJU) for International Students, which assesses academic Japanese skills. Although the levels and types of tests differed, Japanese language proficiency of the participants was ascertained to be sufficiently high for their participation in this experiment. Their Japanese proficiency was verified just before the MRI experiment with the Minimal Test (M-Test;^84^) to certify that they were able to complete the task. At this stage, one non-native participant was excluded due to low score (lower than 2SD from the average within the non-native group) on the Japanese proficiency test. After the elimination, we analyzed the data from 22 non-native participants aged 19–40 years (8 men and 14 women; mean age = 26.8 years; SD = 4.8 years).

All participants were right-handed according to the Edinburgh Handedness Inventory^85^. None of the participant had a history of symptoms requiring neurological, psychological, or other medical care.

### Experimental schedule

The study was conducted over two days for non-native participants, and one day for native participants. We adopted a two-day schedule for non-native participants as they required more time to understand instructions than native speakers. On day 1, the participants took the Japanese proficiency test, and the experimental procedure was explained to them, which helped avoid withdrawal of participation in the actual experiment on the day. The Japanese proficiency test was administered individually in a classroom setting with an examiner present and lasted approximately 3 min. On day 2, the fMRI experiment was conducted. Before the experiment, participants received experimental instruction. The fMRI experiment was divided into three runs.

### Japanese proficiency test

We utilized the Minimal Test of participants’ Japanese proficiency^84^ to certify whether participants would be able to conduct our task. In this test, participants inserted one Japanese hiragana character into a blank space in a sentence, while listening to a compact disc audio recording that narrated the reading passages written on a test sheet; the whole procedure took approximately three minutes. The Minimal Test contained 46 blanks, and the test sentences were created based on grammar items listed in the textbook, Yookoso!^86^. The M-Test has shown correlations with traditionally used placement tests^84^.

## Experimental task

### Stimuli

Ninety Japanese sentences were selected as stimuli from material that was used for teaching Japanese as a second language^83^ and each sentence was used twice in the grammar and letter conditions, respectively (Supplementary Tables S3 and S4). In the grammar condition, participants were required to provide the most appropriate particle to complete the sentence. Seven types of particles containing one mora (one hiragana character) were selected from among case particles, connecting particles, postpositional particles, conjunctive particles, as well as the suffixes of adjectives and adverbs, and parts of adverbs. Some sentences allowed several alternatives as correct answers. For example, both *wa* and *ga* could be used as subject markers. Thus, as long as the sentence was coherent, it was considered correct. In the letter condition, the participants were required to simply read out a mora that was indicated by an underlined space.

### Stimulus presentation

The participants lay in the MRI scanner with plugged ears and foam padding around their heads. We used Presentation software (Neurobehavioral Systems, Albany, CA, USA) to present visual and auditory stimuli and to record button responses. Visual stimuli were projected onto a half-transparent screen with a liquid–crystal display projector (CP-SX12000J; Hitachi Ltd., Tokyo, Japan). The participants viewed stimuli via a mirror placed above the head coil. The viewing angle was sufficiently large for participants to observe stimuli (13.1° [horizontal] × 10.5° [vertical] at maximum). The participants listened to auditory stimuli through ceramic headphones (KIYOHARA-KOUGAKU, Tokyo, Japan). Their utterances were recorded with an opto-microphone system (KOBATEL Corporation, Kanagawa, Japan) and their facial images were recorded with an infrared camera (NAC Image Technology Inc., Tokyo, Japan). The video data were used to calculate the RT and appropriateness of the particle produced.

### MRI data acquisition

We used a 3 T whole-body scanner (Verio; Siemens Erlangen, Germany) with a 32-element phased-array head coil. To obtain T2*-weighted (functional) images in which a multiband echo-planar imaging (EPI) sequence collected multiple EPI slices simultaneously and reduced the volume acquisition time (TA)^87^. We utilized the following sequences to cover the whole brain: repetition time (TR) = 5 s; echo time (TE) = 30 ms; TA = 0.5 s; flip angle (FA) = 90°; field-of-view (FOV) = 192 mm × 192 mm; in-plane resolution = 3 mm × 3 mm; 42 3-mm axial slices with a 17% slice gap; and multiband factor = 6. We utilized a sparse sampling design, in which the production phase was performed during the silences between image acquisitions, to reduce effect of movement. A T1-weighted high-resolution anatomical image was obtained from each participant (TR = 1.8 s; TE = 1.98 ms; FA = 9°; FOV = 256 mm × 256 mm; slice thickness = 1 mm) after the functional imaging runs.

### Task schedule

The participants performed three runs, each lasting 405 s (81 volumes per run). Each run comprised 75 trials of 5 s each (375 s); the grammar and letter conditions were presented 30 times and the null condition, which lasts for 5 s with a fixation, was presented 15 times in each run. We inserted a white cross for 15 s as a baseline before the first trial and for 15 s as a baseline after the last trial (375 + 30 = 405 s). Figure 1 shows the task schedule for each trial. Each trial comprised two phases: preparation and production. In the preparation phase, the predicate of the sentence was visually presented on the screen. At the same time, the stimulus sentence was read aloud by the personal computer (PC). This phase lasted 1300 ms. In the production phase, an argument (subject/object) required by the predicate or an adjunct, which did not conflict with the predicate, was presented followed by a blank with a line that participants used to fill in with the correct particle in the grammar trial.

In the letter trial, the stimulus sentence was presented and contained an underlined letter (hiragana character), indicating participants to read it out. The stimulus sentence was read aloud by the PC while being visually presented on-screen. The audio lasted for approximately 308 to 889 ms. During this phase, the participants were asked to produce the particle as soon as the audio finished. The participants were also required to either produce a particle or read out an underlined letter by the time the next scan started. This phase lasted 3000 ms. The visual stimuli changed into a cross fixation point after 1300 ms.

### Behavioral data analysis

#### Error rate

We checked the error rate of the produced particles and letters read out during the experiment, and double checked them after the experiment from the recorded video. For the analysis, we used 30 sentences for each session to generate 90 sentences in total. Thus, 90 was the maximum score for both the grammar and letter conditions.

#### RT

The RTs of the produced particles and readout letters were measured. The RT was set as the length of time between the end of the PC sound and the onset of particle production/readout letter. Our video system recorded both scanning sound and the participant's voice while scanning. To calculate the RT, we first ordered the two examiners to code the time between the end of the scan sound and the beginning of the participant's voice with Adobe Audition (Adobe Systems Inc., San Jose, CA, USA). Next, we automatically measured the PC sound duration with an in-house script on MATLAB 2016b (MathWorks, Natick, MA, USA). Finally, we determined the RT for each production from the average of times between two examiners by subtracting the two components, the time of previous PC sound before production and the time between the sound and the end of the scan sound (1500 ms, see Fig. 1). The reported time between these two examiners was highly reliable (inter-rater reliability: kappa = 0.94). For the statistical analysis, we transformed the RT for each condition using log transformation and calculated the differential value between them in each group, so that they would approach a normal distribution^88^.

### Statistical analyses

Behavioral data, error rate, and RT were analyzed with linear mixed-effects modelling, which allow for the inclusion of multiple participant-level and stimulus-level independent variables in a single analysis^89^. Analyses were conducted using mixed-effects models with crossed random effects for subjects and items using the lme4 package (version 1.1–23) of R (version 3.6.0). *P*-values were determined using the lmerTest package, which employs the Saitterthwait approximation to compute degrees of freedom for the t-statistic of fixed effects. The analysis included contrast-coded fixed effects for conditions (− 0.5 = Letter, 0.5 = Grammar) and group (− 0.5 = Native, 0.5 = Non-native) in a 2 × 2 factorial design. Random effects were fit using a maximal random-effects structure^90^. This included random intercepts for subjects and items, by subject random slopes for conditions and by-item random slopes for group. Models were fit using a maximum likelihood technique. For analysis, the RT was log transformed after adding one.

### fMRI data analysis

Image processing and statistical analyses were performed using the Statistical Parametric Mapping package (SPM12; Wellcome Trust Centre for Neuroimaging, London, UK). The first functional images were discarded in each run to allow the signal to reach a state of equilibrium. The remaining volumes were used for subsequent analyses. To correct the participants’ head motions, we aligned the functional images from each run to the first image and realigned them to the mean image once again after the first realignment. Each participant’s T1-weighted anatomical image was co-registered with the mean image of all EP images for each participant. The co-registered anatomical image was processed using a unified segmentation procedure combining segmentation, bias correction, and spatial normalization^91^. Using the estimated normalized parameters, all functional images were spatially normalized to the template brain and resampled to a final resolution of 1 × 1 × 1 mm^3^. The normalized EP images were filtered using a Gaussian kernel of 8 mm (full width at half-maximum) in the x, y, and z axes.

Concerning fMRI data analysis, linear contrasts between conditions were calculated for individual participants and incorporated into a random-effects model to make inferences at the population level^92^.

### Initial individual analysis

Following pre-processing, task-related activation was evaluated using a general linear model^93,94^. The design matrix contained regressors of three fMRI runs. Each run included two regressors of interest (grammar and letter) that were modelled at the onsets of the trial. The duration of each regressor was 5000 ms. These conditions were presented in a mixed sequence. Therefore, we adopted the event-related design. The blood-oxygen level-dependent signal for all tasks was modelled with boxcar functions convoluted with the canonical hemodynamic response function. We modelled the task difficulty of grammar processing that differs between native and non-native groups. As task sensitivity is reflected in the error distribution, we added the rate of incorrectness for each syntax within each group as the modulation term on the regressor of the grammar condition. In addition, we only modelled correct trials for each participant to exclude the activation associated with error (results from analysis with all trials were added and are reported in Supplementary Figure S2). Six regressors of rigid-body head motion parameters (three displacements and three rotations) were included as regressors of no interest. Two additional regressors, describing the intensities of the white matter and cerebrospinal fluid were added to the model to account for image-intensity shifts attributable to the movement of the head associated with utterance within the scanner. We also applied a high-pass filter with a cut-off of 128 s to remove low-frequency signal components. Assuming a first-order autoregressive model, we estimated which serial autocorrelation from the pooled active voxels with the restricted maximum likelihood (ReML) procedure to apply to whiten the data^95^. No global scaling was performed. To calculate the estimated parameters, we performed a least-squares estimation on the whitened data. The weighted sum of the parameter estimates in the individual analyses constituted contrast images. The contrast images obtained from the individual analyses represented the normalized task-related increment of the MR signal of each participant. To evaluate the neural substrates involved in processing for grammar production, we compared the mean activation produced by particle production and by letter production in all voxels in the brain (Grammar > Letter).

### Subsequent random-effects analysis

Contrast images from the individual analyses were used for the group analysis. The contrast images obtained from the individual analyses represented the normalized task-related increment of the MRI signal of each participant. In contrast (grammar > letter), a two-sample t-test was performed between non-native and native participants for every voxel in the brain, to obtain population inferences**,** with chronological ages different between the groups (t = 2.02, *p* = 0.049) as a covariate. The resulting set of voxel values for each contrast constituted a statistical parametric map of the t-statistic (SPM {t}).

We evaluated the effects of grammar processing in both groups and the common brain activation between groups as a conjunction of non-native learners and native speakers (conjunction-null hypothesis)^95,96^. We also evaluated whether brain activation was higher in non-native learners than in native speakers for grammar processing as (non-native > native). The SPM{t} threshold was set at t > 3.29 (equivalent to *p* < 0.001 uncorrected). The statistical threshold for the spatial extent test on the clusters was set at *p* < 0.05 and was corrected for multiple comparisons [family-wise error (FWE)] over the whole brain region ^97^.

We evaluated brain activation after excluding any activations outside the gray matter with the explicit masking procedure. Brain regions were anatomically defined and labelled according to Automated Anatomical Labeling^98^, the SUIT template^99^, and an atlas of the human brain^100^.

## Supplementary Information

Supplementary Information.

## Data Availability

The datasets used and/or analyzed during the current study are available from the corresponding author on reasonable request.
