# Supplementary material for: Neural underpinning of Japanese particle processing in non-native speakers
Source: Sci Rep. 2022 Nov 5;12:18740. doi: 10.1038/s41598-022-23382-8 (PMC9637203; doi:10.1038/s41598-022-23382-8)
Supplement: Supplementary file 1 — Supplementary Information. [file 41598_2022_23382_MOESM1_ESM.docx]

**Supplementary Information**

**Title:** Neural underpinning of Japanese particle processing in non-native speakers

Chise Kasai, Motofumi Sumiya, Takahiko Koike, Takaaki Yoshimoto, Hideki Maki, Norihiro Sadato

**Supplementary Information**

Supplementary Table S1. Demographic data for non-native learner.

Supplementary Table S2. Demographic data for native speaker.

Supplementary Table S3. Stimulus sentence.

Supplementary Table S4. Stimulus sentence translated in English.

Supplementary Table S5. Results of linear mixed effects modeling on RT and error rate

Supplementary Figure S1. Behavioral result (log1p RT)

Supplementary Figure S2. Regions associated with grammar processing (modelled all trials including error trials)

Supplementary Table S1. Demographic data for non-native learner.

| ID | Sex | Age | M-Test  (*1) | Academic background  (*2) | First  language | Starting age of Japanese learning | Length of Japanese learning (year)  (*3) | Length of stay in Japan (year) |
| --- | --- | --- | --- | --- | --- | --- | --- | --- |
| F_01 | m | 28 | 45 | 2 | Swedish | 20 | 8 | 2.24 |
| F_02 | m | 27 | 38 | 2 | Chinese | 18 | 4 | 3.5 |
| F_03 | f | 25 | 36 | 2 | Chinese | 19 | 4 | 0.08 |
| F_04 | f | 28 | 46 | 1 | Chinese | 22 | 2.48 | 5.48 |
| F_05 | f | 23 | 42 | 1 | Chinese | 18 | 5 | 0.48 |
| F_06 | f | 24 | 41 | 1 | Chinese | 19 | 4 | 0.48 |
| F_07 | m | 29 | 42 | 2 | Chinese | 26 | 3 | 3 |
| F_08 | m | 24 | 30 | 2 | Chinese | 24 | 2 | 0.48 |
| F_09 | f | 29 | 32 | 2 | Chinese | 25 | 2.48 | 5 |
| F_10 | f | 27 | 35 | 2 | Chinese | 21 | 6 | 3.48 |
| F_11 | m | 34 | 44 | 2 | Mongolian | 22 | 5 | 12 |
| F_12 | m | 28 | 37 | 2 | Mongolian | 21 | 7 | 7 |
| F_13 | f | 35 | 31 | 2 | Mongolian | 25 | 3 | 10 |
| F_14 | f | 28 | 46 | 2 | Mongolian | 21 | 7 | 7 |
| F_15 | f | 40 | 39 | 2 | Mongolian | 24 | 16 | 15 |
| F_16 | m | 26 | 39 | 1 | Chinese | 24 | 2 | 1.56 |
| F_17 | f | 24 | 41 | 1 | Korean | 15 | 5 | 1 |
| F_18 | f | 21 | 45 | 1 | Chinese | 18 | 3 | 3 |
| F_19 | f | 19 | 43 | 1 | Chinese | 15 | 4 | 2 |
| F_20 | f | 26 | 35 | 1 | Chinese | 25 | 1 | 0.48 |
| F_21 | m | 23 | 44 | 1 | Chinese | 19 | 2 | 2.32 |
| F_22 | f | 21 | 41 | 1 | Malay | 19 | 2.48 | 3.48 |
| F_23 | m | 44 | 25 | 1 | Swedish | 19 | 8 | 5 |

*1: Minimal Test (M-Test), full score = 46 points

*2: Academic background, 0 = not certain, 1 = Bachelor, 2 = Master of Arts or Science, 3 = Doctor of Philosophy

*3: The participants learned Japanese in their home countries through school education and self-learning and continued their study in Japanese universities.

Supplementary Table S2. Demographic data for native speaker.

| ID | Sex | Age | M-Test  (*1) | Academic background  (*2) | First language |
| --- | --- | --- | --- | --- | --- |
| J_01 | m | 21 | 44 | 1 | Japanese |
| J_02 | m | 22 | 45 | 1 | Japanese |
| J_03 | m | 21 | 46 | 1 | Japanese |
| J_04 | m | 25 | 46 | 2 | Japanese |
| J_05 | m | 21 | 43 | 1 | Japanese |
| J_06 | m | 20 | 46 | 1 | Japanese |
| J_07 | m | 36 | 46 | 3 | Japanese |
| J_08 | f | 39 | 44 | 3 | Japanese |
| J_09 | m | 21 | 45 | 1 | Japanese |
| J_10 | f | 19 | 45 | 1 | Japanese |
| J_11 | m | 21 | 45 | 1 | Japanese |
| J_12 | f | 18 | 45 | 1 | Japanese |
| J_13 | f | 18 | 46 | 1 | Japanese |
| J_14 | f | 21 | 46 | 1 | Japanese |
| J_15 | f | 21 | 46 | 1 | Japanese |
| J_16 | f | 20 | 46 | 1 | Japanese |
| J_17 | m | 21 | 43 | 1 | Japanese |
| J_18 | m | 32 | 43 | 3 | Japanese |
| J_19 | m | 24 | 46 | 1 | Japanese |
| J_20 | f | 26 | 41 | 1 | Japanese |
| J_21 | f | 28 | 42 | 1 | Japanese |
| J_22 | f | 26 | 46 | 1 | Japanese |
| J_23 | m | 24 | 46 | 2 | Japanese |
| J_24 | m | 28 | 46 | 0 | Japanese |
| J_25 | f | 19 | 45 | 1 | Japanese |

*1: Minimal Japanese Test (full score = 46 points)

*2: Academic background, 0 = not certain, 1 = Bachelor, 2 = Master of Arts or Science, 3 = Doctor of Philosophy

Supplementary Table S3. Stimulus sentence.

|  | the predicate | the subject | particles |
| --- | --- | --- | --- |
| 1 | くれましたか | だれ | が |
| 2 | くれました | ちち | が |
| 3 | ふっています | あめ | が |
| 4 | できましたよ | ごはん | が |
| 5 | ついています | テレビ | が |
| 6 | いいですか | どちら | が |
| 7 | ながいです | あし | が |
| 8 | いたいです | あたま | が |
| 9 | おおいですね | くるま | が |
| 10 | たかいです | くるま | は |
| 11 | さむかったですね | きのう | は |
| 12 | あつかったですか | ロンドン | は |
| 13 | いいです | あたま | が/は |
| 14 | すてきですね | デザイン | が/は |
| 15 | たのしみです | たべるの | が/は |
| 16 | どちらですか | おたく | は |
| 17 | どちらですか | たなかさん | は |
| 18 | なんですか | あれ | は |
| 19 | いくらですか | これ | は |
| 20 | どこのですか | そのカメラ | は |
| 21 | いかがでしたか | パリ | は |
| 22 | いかがですか | コーヒー | は |
| 23 | いけませんか | たべて | は |
| 24 | ありがとう | せんじつ | は |
| 25 | ははです | わたし | の/は/が |
| 26 | カメラです | ちち | の |
| 27 | ほんです | りょうり | の |
| 28 | すきですか | どんなひと | が |
| 29 | すきです | バナナ | が/は |
| 30 | すきですか | どれ | が |
| 31 | ほしいです | デジカメ | が |
| 32 | ほしいです | くるま | が |
| 33 | みせてください | メニュー | を |
| 34 | かいましたか | なに | を |
| 35 | つけてください | テレビ | を |
| 36 | つれていきます | こども | を |
| 37 | ください | こうちゃ | を |
| 38 | たべませんか | ケーキ | を |
| 39 | かざりましょう | しゃしん | を |
| 40 | あけてください | ドア | を |
| 41 | つけてください | テレビ | を |
| 42 | あるきます | みち | を |
| 43 | あるきましょう | みぎがわ | を |
| 44 | あげました | いもうと | に |
| 45 | のせてあげました | くるま | に |
| 46 | もらいました | ともだち | に |
| 47 | いきましたか | なんがつ | に |
| 48 | しましたか | なんようび | に |
| 49 | いきました | すいようび | に |
| 50 | かいました | かようび | に |
| 51 | たべます | たま | に |
| 52 | はじまります | ９じ | に |
| 53 | もどります | ひるまで | に |
| 54 | すんでいます | パリ | に |
| 55 | つとめています | かいしゃ | に |
| 56 | すんでいます | ここ | に |
| 57 | とまっています | ホテル | に |
| 58 | かいました | パリ | で |
| 59 | たべたいです | ホテル | で |
| 60 | ねています | へや | で |
| 61 | かいましたか | どこ | で |
| 62 | およぎます | プール | で |
| 63 | たべます | うち | で |
| 64 | たべます | うち | で |
| 65 | たべます | へや | で |
| 66 | たべましょう | レストラン | で |
| 67 | たべます | はし | で |
| 68 | きました | バス | で |
| 69 | いきました | バス | で |
| 70 | いくらですか | ぜんぶ | で |
| 71 | おなじです | あべさん | と/に |
| 72 | あそびにきます | ともだち | と |
| 73 | いきたいですか | だれ | と |
| 74 | いきたいですか | だれ | と |
| 75 | いきたいですね | どこ | か |
| 76 | たべたいですね | なに | か |
| 77 | たべましょうか | なに | か |
| 78 | ありません | いちど | も |
| 79 | やさしいです | しんせつ | で |
| 80 | やすいです | おいしく | て |
| 81 | ひとですか | がんこ | な |
| 82 | ところです | すてき | な |
| 83 | ひとです | ゆうめい | な |
| 84 | さむくありません | そんな | に |
| 85 | よくありません | そんな | に |
| 86 | よくないです | そんな | に |
| 87 | あらいましょう | きれい | に |
| 88 | なりました | べんり | に |
| 89 | なりました | あたたか | く |
| 90 | なりました | いくこと | に |

*1: Before the experiment, we conducted a pilot study to examine how Japanese native speakers would respond to the stimuli sentences. The instruction given was to choose the most appropriate particles. Although different answers were found for six stimuli sentences (13, 14, 15, 25, 29, and 71 in Supplementary Information), the rest had consistent answers. Therefore, we considered the pilot results as correct answers, and for the six stimuli, we responded flexibly; if one of the alternatives was chosen, it was regarded as a correct answer.

Supplementary Table S4. Stimulus sentence translated in English.

|  | **The predicate** | **The subject** | **case particle** | **Intended meaning** |
| --- | --- | --- | --- | --- |
| 1 | gave (it) | who | ga | Who gave (it to you)? |
| 2 | gave (it) | my father | ga | My father gave (it to me). |
| 3 | falling | rain | ga | It is raining. |
| 4 | be ready | meal | ga | The meal is ready. |
| 5 | be on | TV | ga | The TV set is on. |
| 6 | do (you) prefer | which | ga | Which do you prefer? |
| 7 | be long | legs | ga | You have long legs. |
| 8 | hurt | head | ga | I have a headache. |
| 9 | (there are) many | cars | ga | There are many cars. |
| 10 | be expensive | cars | wa | Cars are expensive. |
| 11 | was cold | yesterday | wa | It was cold yesterday. |
| 12 | was hot | (in) London | wa | Was it hot in London? |
| 13 | be good | head | ga/wa | (S/he) is smart. |
| 14 | be nice | the design | ga/wa | The design is nice. |
| 15 | be looking forward to | eating | ga/wa | (I ) am looking forward to eating (it). |
| 16 | where | your house | wa | Where is your house? |
| 17 | which is | Ms./Mr. Tanaka | wa | Which is Ms./Mr. Tanaka? |
| 18 | what is | that | wa | What is that? |
| 19 | how much is | this | wa | How much is this? |
| 20 | which make is | the camera | wa | Which make is the camera? |
| 21 | how was | Paris | wa | How was Paris? |
| 22 | what about | coffee | wa | How about coffee? |
| 23 | be not good | eat | wa | Is it not good to eat? |
| 24 | thank you | the other day | wa | Thank you for the other day. |
| 25 | be (someone's) mother | my | no/wa/ga | (This) is my mother. |
| 26 | be a camera | father | no | (This) is my father's camera. |
| 27 | be a book | cookery | no | (This) is a cook book. |
| 28 | do (you) like | what person | ga | What persons do you like? |
| 29 | like | banana | ga/wa | (I) like bananas. |
| 30 | do (you) like | which | ga | Which do (you) like? |
| 31 | want | a digital camera | ga | (I) want a digital camera. |
| 32 | want | a car | ga | (I) want a car. |
| 33 | show (me) | the menu | o | Please show (me) the menu. |
| 34 | bought | what | o | What did (you) buy? |
| 35 | turn on | TV | o | Please turn on the TV. |
| 36 | will take | children | o | (I) will take (my) children. |
| 37 | give (me) | tea | o | Please give (me) some tea. |
| 38 | shall (we) eat | cake | o | Shall (we) eat cake? |
| 39 | let's decorate | a photo | o | Let's put photos (somewhere). |
| 40 | please open | the door | o | Please open the door. |
| 41 | turn on | TV | o | Please turn on the TV. |
| 42 | walk | road | o | (We) walk on the road. |
| 43 | let us walk | the right side | o | Let us walk on the right side. |
| 44 | gave | sister | ni | (I) gave (it) to my sister. |
| 45 | gave a lift | car | ni | (I) gave a lift (to someone). |
| 46 | received | friend | ni | (I) received (something) from my friend. |
| 47 | did (you) go | what month | ni | What month did (you) go? |
| 48 | did (you) do | which day of the week | ni | Which day of the week did (you) do (it)? |
| 49 | went | Wednesday | ni | (I) went (there) on Wednesday. |
| 50 | bought | Tuesday | ni | (I) bought (it) on Tuesday. |
| 51 | eat | sometimes | ni | (I) sometimes eat (it). |
| 52 | (it) starts | nine o'clock | ni | (It) starts at nine o'clock. |
| 53 | will return | till noon | ni | (I) will return till noon. |
| 54 | live | Paris | ni | (I) live in Paris. |
| 55 | work | a company | ni | (I) am working for a company. |
| 56 | live | here | ni | (I) live here. |
| 57 | (be) staying | a hotel | ni | (I) am staying at (this) hotel. |
| 58 | bought | Paris | de | (I) bought (it) in Paris. |
| 59 | want to eat | a hotel | de | (I) want to eat (It) at the hotel. |
| 60 | be sleeping | a room | de | (I) am sleeping in the room. |
| 61 | did (you) buy | where | de | Where did (you) buy (it)? |
| 62 | swim | a swimming pool | de | (I) swim in the swimming pool. |
| 63 | eat | home | de | (I) eat at home. |
| 64 | eat | home | de | (I) eat at home. |
| 65 | eat | room | de | (I) eat in the room. |
| 66 | let us eat | restaurant | de | Let us eat at the restaurant. |
| 67 | eat | chopsticks | de | (I) eat with chopsticks. |
| 68 | came | bus | de | (I) came (here) by bus. |
| 69 | went | bus | de | (I) went (there) by bus. |
| 70 | how much | all | de | How much is the total? |
| 71 | the same | Ms./Mr. Abe | to/ni | (I have) the same (idea) with Ms./Mr. Abe |
| 72 | come to play | friend | to | (I) come (here) with my friends to play. |
| 73 | do (you) want to go | who | to | Who do (you) want to go with? |
| 74 | do (you) want to go | where | to | Who do (you) want to go with? |
| 75 | want to go | somewhere | ka | (I) want to go somewhere. |
| 76 | want to eat | something | ka | (I) want to eat something. |
| 77 | shall we eat | something | ka | Shall (we) eat something? |
| 78 | have not (done) | never | mo | (I) have never (done it before). |
| 79 | be gentle | kind | de | (That person) is kind and gentle. |
| 80 | be cheap | delicious | te | (That) is delicious and cheap. |
| 81 | be person | stubborn | na | Is (s/he) a stubborn person? |
| 82 | be a place | nice | na | (This) is a nice place. |
| 83 | be a person | famous | na | (S/he) is a famous person. |
| 84 | be not cold | that | ni | (It) is not that cold. |
| 85 | be not good | that | ni | (It) is not that good. |
| 86 | be not good | that | ni | (It) is not that good. |
| 87 | let us wash | clean | ni | Let us wash (it) clean. |
| 88 | became | convenient | ni | (It) became convenient. |
| 89 | became | warm | ku | (It) became warm. |
| 90 | came to | go | ni | (I) have come to go (there). |

Supplementary Table S5: Results of linear mixed effects modeling on RT and error rate

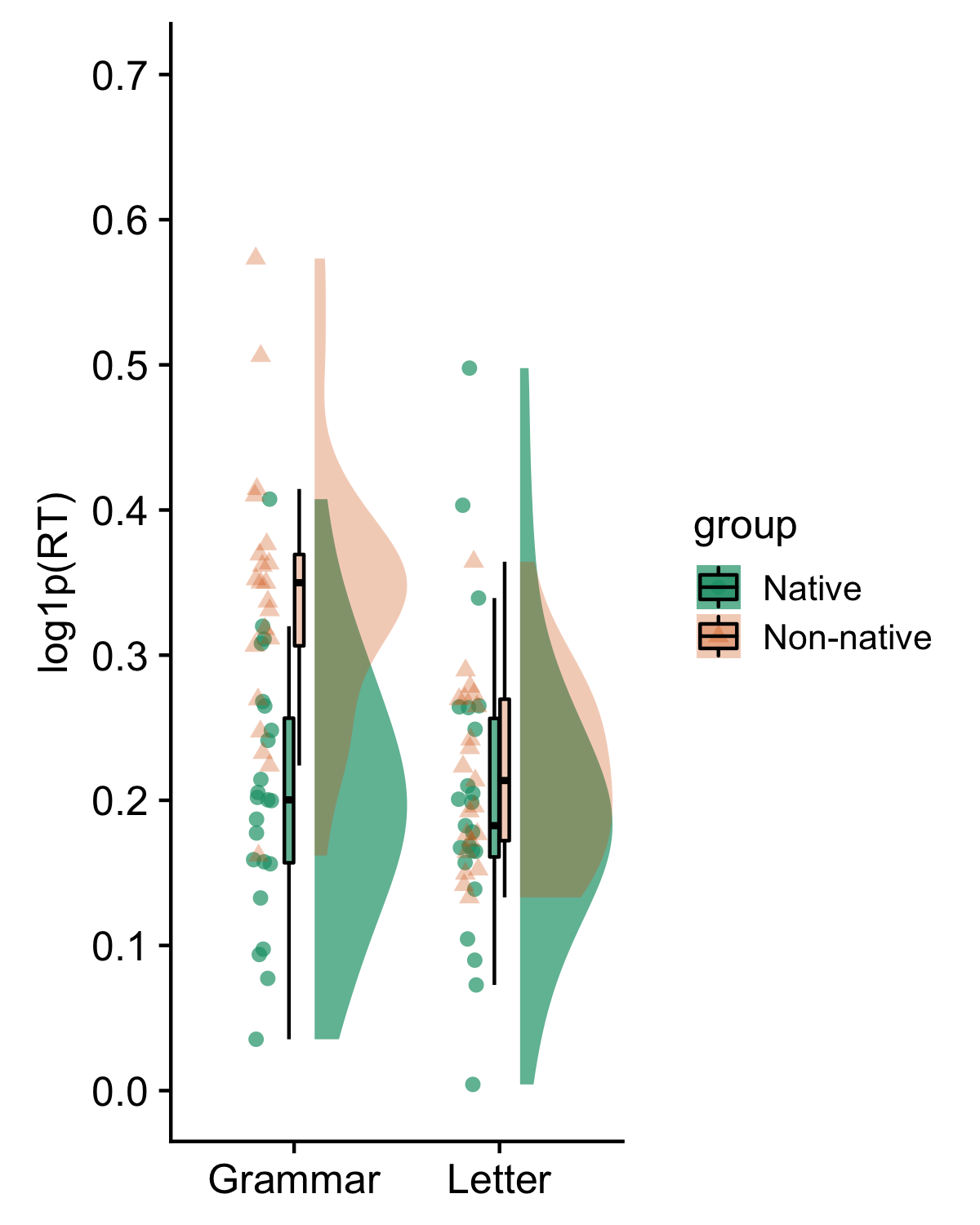


Supplementary Figure S1: Behavioral result (log1p RT)


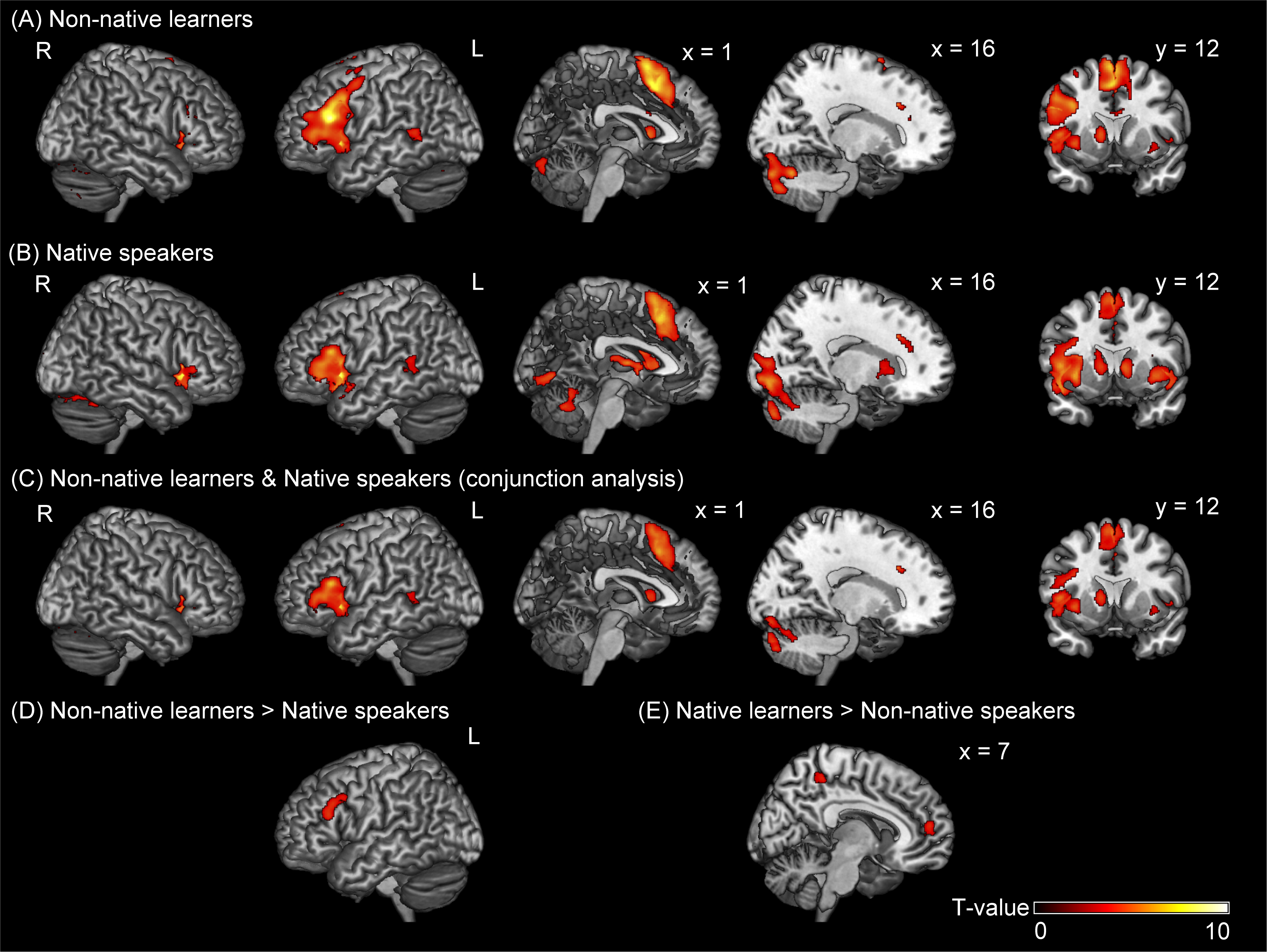


Supplementary Figure S2: Regions associated with grammar processing (modelled all trials including error trials)
